# A CT-based nomogram for predicting the risk of adenocarcinomas in patients with subsolid nodule according to the 2021 WHO classification

**DOI:** 10.1186/s40644-022-00483-1

**Published:** 2022-09-05

**Authors:** Qilong Song, Biao Song, Xiaohu Li, Bin Wang, Yuan Li, Wu Chen, Zhaohua Wang, Xu Wang, Yongqiang Yu, Xuhong Min, Dongchun Ma

**Affiliations:** 1 Department of Radiology, Anhui Chest Hospital, Hefei, China; 2 Clinical College of Chest, Anhui Medical University, Hefei, China; 3 Department of Radiology, the First Affiliated Hospital of Anhui Medical University, Hefei, China; 4 Department of Thoracic Surgery, Anhui Chest Hospital, Hefei, China

**Keywords:** Nomogram model, Lung cancer, CT features, 2021 WHO classification

## Abstract

**Purpose:**

To establish a nomogram for predicting the risk of adenocarcinomas in patients with subsolid nodules (SSNs) according to the 2021 WHO classification.

**Methods:**

A total of 656 patients who underwent SSNs resection were retrospectively enrolled. Among them, 407 patients were assigned to the derivation cohort and 249 patients were assigned to the validation cohort. Univariate and multi-variate logistic regression algorithms were utilized to identity independent risk factors of adenocarcinomas. A nomogram based on the risk factors was generated to predict the risk of adenocarcinomas. The discrimination ability of the nomogram was evaluated using the concordance index (C-index), its performance was calibrated using a calibration curve, and its clinical significance was evaluated using decision curves and clinical impact curves.

**Results:**

Lesion size, mean CT value, vascular change and lobulation were identified as independent risk factors for adenocarcinomas. The C-index of the nomogram was 0.867 (95% CI, 0.833-0.901) in derivation cohort and 0.877 (95% CI, 0.836-0.917) in validation cohort. The calibration curve showed good agreement between the predicted and actual risks. Analysis of the decision curves and clinical impact curves revealed that the nomogram had a high standardized net benefit.

**Conclusions:**

A nomogram for predicting the risk of adenocarcinomas in patients with SSNs was established in light of the 2021 WHO classification. The developed model can be adopted as a pre-operation tool to improve the surgical management of patients.

**Supplementary Information:**

The online version contains supplementary material available at 10.1186/s40644-022-00483-1.

## Keypoints

- The performance and stability of the model was confirmed using internal and external validation cohorts
- Preoperative CT features can guide surgical intervention or conservative screening
- A nomogram incorporating simple and intuitive CT features was developed for clinical use.

## Introduction

Lung cancer is ranked among the top leading causes of cancer deaths worldwide [1]. In most cases, lung adenocarcinoma (LUAD) is the most prevalent histological subtype of lung cancer accounting for approximately 50%, followed by squamous cell carcinoma, small cell carcinoma, and large cell carcinoma [2]. Currently, early surgical intervention is the most effective treatment for early LUAD [3]. However, cancer overdiagnosis and overtreatment have been shown to increase the demand for healthcare resources. Therefore, identifying strategies for avoiding these challenges may result in better clinical management of patients. Using the next generation sequencing (NGS) technology, scientists have reported that the progression of LUAD involves many steps. For instance, invasive adenocarcinoma (IAC) develops sequentially from atypical adenomatous hyperplasia (AAH) and progresses to adenocarcinoma in situ (AIS), to form a minimally invasive adenocarcinoma (MIA) [4, 5]. In the revised 2021 classification of thoracic tumors, WHO includes AAH and AIS as precursor glandular lesions and classifies MIA and IAC as adenocarcinomas [6]. In the last decade, large-scale, systematic studies have shown that the long-term postoperative disease-specific survival of AIS and MIA may reach 100% [5, 7, 8]. The good long-term survival of patients with MIA indicates that surgical interventions may not benefit patients with AAH or AIS. Therefore, AAH or AIS are mainly managed conservatively and rarely require surgical intervention.

A nomogram has been widely used as a reliable and robust tool to create a visualized graph of a predictive model comprising the risk factors of a clinical event [9]. Some studies have demonstrated that nomogram models can improve disease diagnosis [10, 11]. To date, few nomograms for predicting the risk of adenocarcinomas in patients with subsolid nodule (SSN) in light of the 2021 WHO classification have been reported. In this study, we used pre-operative computed tomography (CT) features to predict the probability of adenocarcinomas in patients with SSN according to the 2021 WHO classification. The developed nomogram is expected to help clinicians make better decisions regarding the surgical management of patients.

## Materials and methods

### Patients

**Fig. 1:**
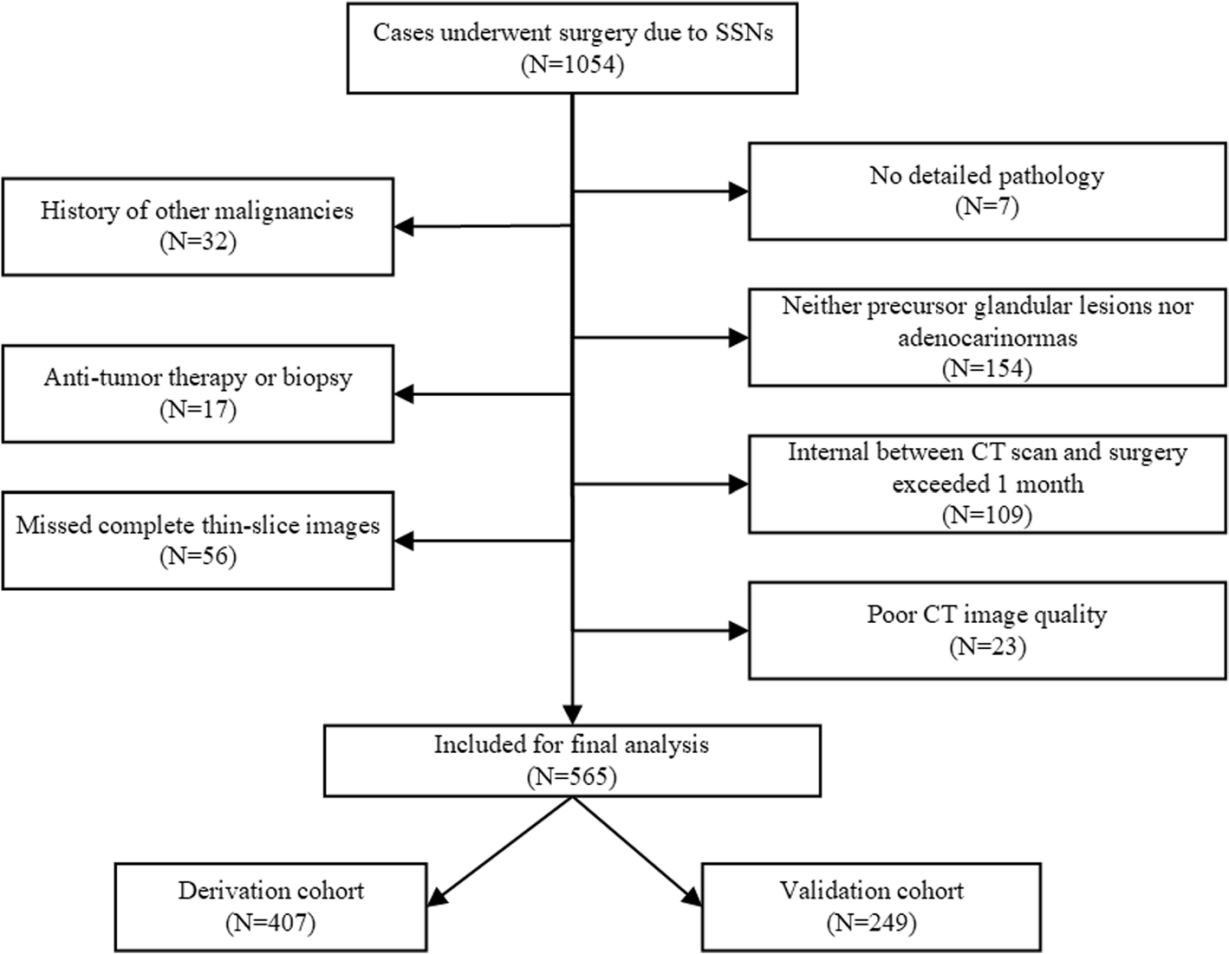
A flowchart showing the patient selection process

A total of 1054 patients with SSNs who underwent surgery at our institution between April 2019 and December 2020 were retrospectively analyzed. All cases were enrolled based on a strict inclusion and exclusion criteria. The inclusion criteria were as follows: (1) patients with SSNs on CT scan; (2) maximum diameter ≤ 30 mm; (3) complete surgical resection. Exclusion criteria were: (1) no detailed pathology; (2) history of other malignancies; (3) neither precursor glandular lesions or adenocarcinomas; (4) anti-tumor therapy or biopsy; (5) CT scan and surgery were separated by more than one month; (6) missed complete thin-slice images (1 mm); (7) poor CT image quality. Finally, 656 cases were enrolled in this study. A flowchart showing participant recruitment is shown in Fig. 1. All clinical characteristics CT images of the participants were extracted from the Picture and Communication Systems (PACS) and the hospital’s electronic medical records (EMR) systems. The Ethics Committee of our institution approved the study (approval No. k2020-009) and waived the requirement of informed consent.

### CT scan protocol

Chest CT images were acquired using a 64-slice spiral CT system (SOMATOM Definition AS+; Siemens Healthcare, Germany) using a pulmonary window center of -500 Hounsfield units (HU), a window width of 1500 HU, mediastinal window center of 50 HU, and a window width of 350 HU. Prior to examination, participants were trained on how to take a deep breath and hold their breath. Each participant was scanned while in the headfirst supine position, with arms raised. CT scans were acquired at the end of inspiration, with the CT scanning ranging from the tip of the lung to the diaphragm. Scanning parameters were as follows: 120 kVp tube voltage, automatic tube current modulation, a pitch of 1.2, a matrix of 512×512, 7 mm slice thickness, and reconstructed slice thickness of 1 mm with high-resolution reconstruction algorithm.

### Imaging analysis

CT images were examined by radiologists A, B and C (with 4, 9 and 20 years of chest CT imaging experience, respectively), using a commercially available advanced workstation (VE40A, Siemens Healthineers) containing a tumor quantitative analysis software package (Siemens Healthineers). All radiologists were blinded to all clinical data and pathological results, except for age and sex of the patients. The software package has a semi-automated lesion segmentation tool. Raw 1 mm CT imaging data (DICOM format) were loaded into the software package. SSNs were segmented manually by tracing the segmentation boundaries of the nodules slice by slice, on axial images, excluding areas where large vessels and bronchi predominated. Nodule segmentation was conducted by radiologist A using the semi-automated tool. Segmentation boundaries were checked by radiologist B, and any discrepancies of segmentation boundaries of SSNs between the two radiologists were resolved by radiologist C. The segmentation tool produced long axis, short axis, mean CT value, and volume of the lesion. Lesion size was calculated as the average of the long- and short-axis diameters. Nodule mass was calculated as using the formula: mass(g) = (mean CT value + 1000) × volume(cm^3^)/1000 [12]. Morphological features of the SSNs were examined by radiologists A and B through multiple planar reconstruction (MPR), maximal intensity projection (MIP), volume rending technique (VRT), and minimum intensity projection (MinIP). The morphological features of SSNs including the following: (1) vascular change was abnormal vascular broadening or distortion [13], and was classified as present or absent; (2) bronchiole change was bronchus with dilated or tortuous lumen [14], and was classified as present or absent; (3) lobulation was defined as the outline of the lesion is not purely circular or oval, was classified as present or absent; (4) bubble was defined as a gaseous density with maximum diameter < 5mm [15], was classified as present or absent; (5) pleural attachment was defined as the pleura that was pulled to the lesion by a linear structure, and was classified as present or absent; (6) spiculation was defined as linear strands extending beyond the lesion [16], and was classified as present or absent; (7) lesion-lung interface, the border between the lesion and the normal lung tissues, and was classified as clear and blurry. Any discrepancies in the morphological features of SSNs between radiologists A and B were resolved by radiologist C.

### Interobserver and intraobserver agreements

Agreements between radiologists A, B, and C were evaluated with the Intraclass correlation coefficient (ICC) for quantitative parameters and Kappa coefficient for categorical variables. 30 SSNs (5%) were randomly selected for segmentation and morphological feature evaluation. At first, radiologists A, B, and C conducted segmentation and morphological feature evaluation independently. After 2 weeks, the segmentation and morphological feature evaluation of the SSNs were performed by three radiologists a second time.

### Pathological diagnosis

All enrolled pathological specimens were independently reviewed by two senior pathologists, and the final pathological results were obtained through consensus. Pathological diagnosis was classified as AAH, AIS, MIA, and IAC based on the 2015 WHO classification of pulmonary adenocarcinomas [4]. Notably, AAH and AIS were classified as precursor glandular lesions and MIA and IAC as adenocarcinomas based on the 2021 WHO classification [6].

### Statistical analysis

SPSS 26.0 (IBM) and R 3.5.1 (http://www.r-project.org) were used for statistical analyses. Normality of quantitative parameters was tested using the Shapiro-Wilk test. Quantitative parameters satisfying the normal distribution were expressed as ‾x ± s. Otherwise, quantitative parameters were expressed as median (P25, P75). Student’s t test or Mann-Whitney U test were used to compare differences between continuous data. Categorical variables were compared using the chi-square test. Univariate and multivariate analyses using logistic regression were performed for the derivation cohort to identify independent risk factors for adenocarcinoma. The independent risk factors were then used to construct a nomogram. The nomogram’s discriminative capacity was first internally validated using 1,000 bootstrap samples to acquire a Harrell concordance index (C-index) in the derivation cohort. The nomogram was then tested on the validation cohort for external validation. Two-sided *P* <0.05 indicated statistical significance.

## Results

### Demographics of the study cohort

**Fig. 2:**
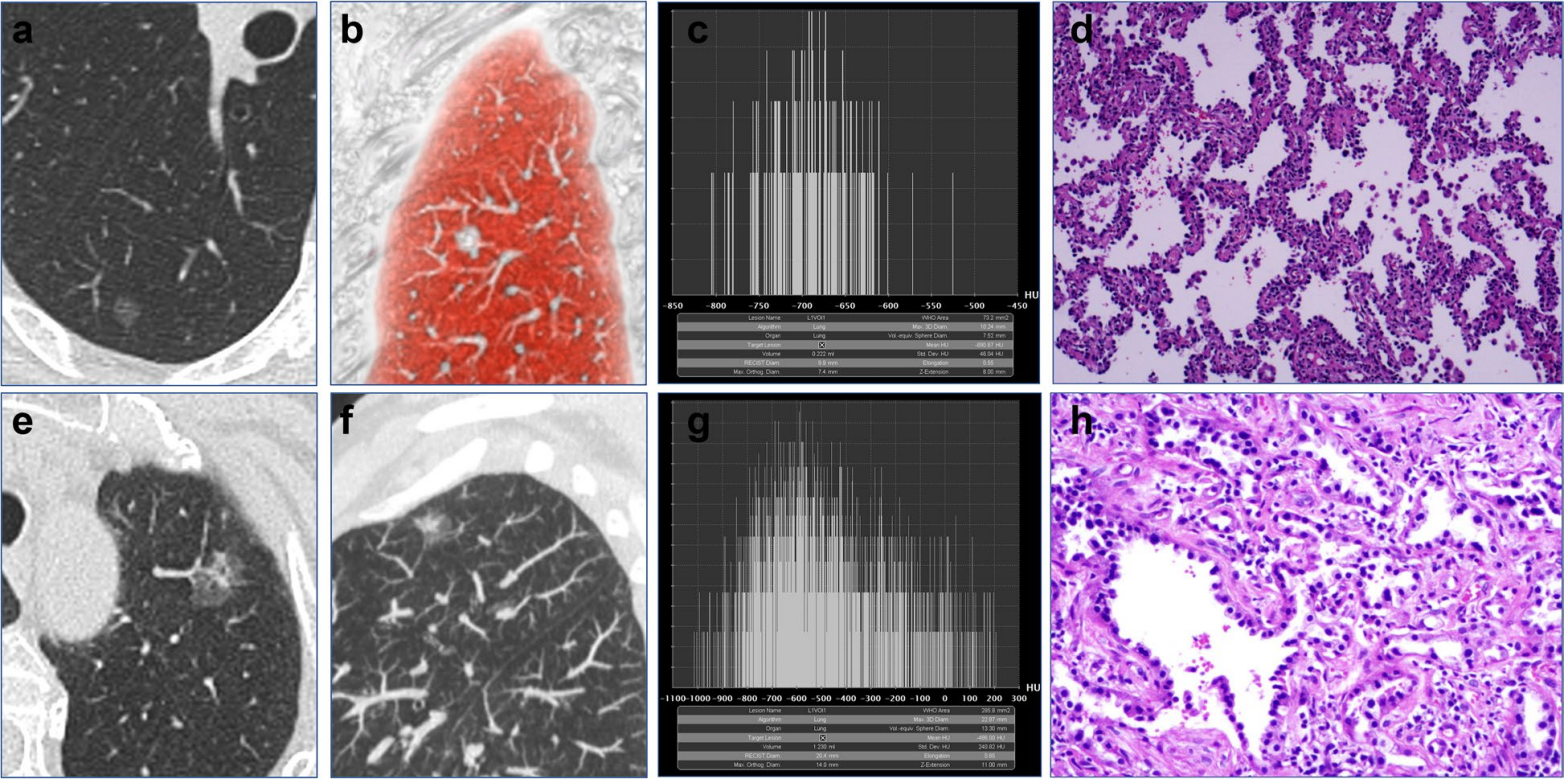
**a-d** CT and pathological images of one adenocarcinoma in situ (AIS) appearing as subsolid nodule (SSN). **a** CT multiplanar reconstruction (MPR) and **b** volume rending technique (VRT) showing the absence of vascular change, and lobulation (axial and coronal). **c** The long axis, short axis, and mean CT value were calculated using the semi-automated segmentation tool. **d** Pathology: the tumor cells were attached to the alveolar wall; the basement membrane was intact. (HE staining ×100). **e-h** CT and pathological images of one invasive adenocarcinoma (IAC) appearing as SSN. **e** CT MPR and **f** maximal intensity projection (MIP) showing the presence of vascular change, and lobulation (axial and sagittal). **g** The long axis, short axis, and mean CT value were calculated using the semi-automated segmentation tool. **h** Pathology: the tumor cells damaged alveolar cells, a large number of tumor cells infiltrating the interstitium. (HE staining ×100)

A total of 656 participants (681 SSNs) were enrolled, including 241 males and 415 females, with a mean age of 52.03 ± 12.26. There were 546 never smokers and 110 current or former smokers. Moreover, 407 patients (423 SSNs) operated between April 2019 and April 2020 were into the derivation cohort and 249 patients (258 SSNs) operated between May 2020 and December 2020 were assigned into the validation cohort. The baseline clinical characteristics and CT features of SSNs are shown in Tables 1 and 2, respectively. In derivation cohort, 110 SSNs were diagnosed as precursor glandular lesions (AAH =7, AIS =103), and 313 SSNs were diagnosed as adenocarcinomas (MIA =144, IAC =169). There were significant differences between precursor glandular lesions and adenocarcinomas subgroups in the lesion size (*P* <0.001), mean CT value (*P* <0.001), volume (*P* <0.001), mass (*P* <0.001), vascular change (*P* <0.001), bronchiole change (*P* <0.001), lobulation (*P* <0.001), pleural attachment (*P* <0.001), spiculation (*P* =0.032), and lesion-lung interface (*P* <0.001), except for bubble (*P* =0.081). In validation cohort, 72 SSNs were diagnosed as precursor glandular lesions (AAH =4, AIS =68), and 186 SSNs were diagnosed as adenocarcinomas (MIA =89, IAC =97). There were significant differences between precursor glandular lesions and adenocarcinomas subgroups in the lesion size (*P* <0.001), mean CT value (*P* <0.001), volume (*P* <0.001), mass (*P* <0.001), vascular change (*P* <0.001), bronchiole change (*P* =0.003), lobulation (*P* <0.001), pleural attachment (*P* =0.006), lesion-lung interface (*P* =0.003), except for bubble (*P* =0.359), and spiculation (*P* =0.092). The CT features and pathological results are shown in Table 3. The CT and pathological images from the 2 examples are shown in Fig. 2.

**Table 1:** Baseline clinical characteristics of patients with SSNs

**Characteristics**	**Total (*N*=656)**	**Derivation cohort (*N*=407)**	**Validation cohort (*N*=249)**	**t/χ**^**2**^	***P* value**
Age	52.03 ± 12.26	51.94 ± 12.13	52.17 ± 12.49	-0.231^a^	0.818
Sex				0.171^b^	0.679
Male	241 (36.7%)	152 (37.3%)	89 (35.7%)		
Female	415 (63.3%)	255 (62.7%)	160 (64.3%)		
Smoking history				1.277^b^	0.258
Never smoker	546 (83.2%)	344 (84.5%)	202 (81.1%)		
Current or former smoker	110 (16.8%)	63 (15.5%)	47 (18.9 %)		

^a^t value; ^b^χ^2^ value

**Table 2:** CT features of SSNs in the derivation and validation cohorts

**Characteristics**	**Total (*N*=681)**	**Derivation cohort (*N*=423)**	**Validation cohort (*N*=258)**	**t/Z/χ**^**2**^	***P* value**
Lesion Size (mm)	11.32 ± 5.00	11.33 ± 4.98	11.30 ± 5.03	0.074^a^	0.941
Mean CT value (HU)	-526.16 ± 130.92	-528.38 ± 131.66	-522.52 ± 129.88	-0.566^a^	0.571
Volume (cm^3^)	0.34 (0.20, 0.82)	0.34 (0.22, 0.82)	0.34 (0.19, 0.80)	0.099^b^	0.921
Mass (g)	0.15 (0.09, 0.34)	0.15 (0.09, 0.35)	0.15 (0.08, 0.33)	-0.163^b^	0.870
Vascular change				0.088^c^	0.767
Present	246 (36.1%)	151 (35.7%)	95 (36.8%)		
Absent	435 (63.9%)	272 (64.3%)	163 (63.2%)		
Bronchiole change				0.357^c^	0.550
Present	132 (19.4%)	79 (18.7%)	53 (20.5%)		
Absent	549 (80.6%)	344 (81.3%)	205 (79.5%)		
Lobulation				0.103^c^	0.748
Present	264 (38.8%)	162 (38.3%)	102 (39.5%)		
Absent	417 (61.2%)	261 (61.7%)	156 (60.5%)		
Bubble				0.718^c^	0.397
Present	97 (14.2%)	64 (15.1%)	33 (12.8%)		
Absent	584 (85.8%)	359 (84.9%)	225 (87.2%)		
Pleural attachment				0.771^c^	0.380
Present	233 (34.2%)	150 (35.5%)	83 (32.2%)		
Absent	448 (65.8%)	273 (64.5%)	175 (67.8%)		
Spiculation				0.051^c^	0.822
Present	111 (16.3%)	70 (16.5%)	41 (15.9%)		
Absent	570 (83.7%)	353 (83.5%)	217 (84.1%)		
Lesion-lung interface				0.568^c^	0.451
Clear	450 (66.1%)	275 (65.0%)	175 (67.8%)		
Blurry	231 (33.9%)	148 (35.0%)	83 (32.2%)		

^a^t value; ^b^Z value; ^c^χ^2^ value

**Table 3:** CT features and pathological results of SSNs in derivation and validation cohorts

**Characteristics**	**Derivation cohort (*N*=423)**				**Validation cohort (*N*=258)**			
	**Precursor glandular lesions (*N*=110)**	**Adenocarcinomas (*N*=313)**	**t/Z/χ**^**2**^	***P* value**	**precursor glandular lesions (*N*=72)**	**Adenocarcinomas (*N*=186)**	**t/Z/χ**^**2**^	***P* value**
Lesion Size (mm)	8.25 ± 1.96	12.41 ± 5.27	-11.824^a^	<0.001	8.09 ± 1.90	12.54 ± 5.31	-9.899^a^	<0.001
Mean CT value (HU)	-603.38 ± 93.89	-502.02 ± 132.98	-8.671^a^	<0.001	-592.19 ± 89.64	-495.54 ± 133.15	-6.719^a^	<0.001
Volume (cm^3^)	0.24 (0.14, 0.38)	0.43 (0.25, 1.05)	-7.325^b^	<0.001	0.20 (0.13, 0.31)	0.45 (0.24, 1.02)	-6.566^b^	<0.001
Mass (g)	0.09 (0.05, 0.15)	0.21 (0.11, 0.48)	-8.919^b^	<0.001	0.08 (0.05, 0.13)	0.21 (0.11, 0.48)	-7.688^b^	<0.001
Vascular change			70.399^c^	<0.001			49.756^c^	<0.001
Present	3 (2.7%)	148 (47.3%)			2 (2.8%)	93 (50.0%)		
Absent	107 (97.3%)	165 (52.7%)			70 (97.2%)	93 (50.0%)		
Bronchiole change			14.838^c^	<0.001			9.121^c^	0.003
Present	7 (6.4%)	72 (23.0%)			6 (8.3%)	47 (25.3%)		
Absent	103 (93.6%)	241 (77.0%)			66 (91.7%)	139 (74.7%)		
Lobulation			75.580^c^	<0.001			48.237^c^	<0.001
Present	4 (3.6%)	158 (50.5%)			4 (5.6%)	98 (52.7%)		
Absent	106 (96.4%)	155 (49.5%)			68 (94.4%)	88 (47.3%)		
Bubble			3.047^c^	0.081			0.843^c^	0.359
present	11 (10.0%)	53 (16.9%)			7 (9.7%)	26 (14.0%)		
Absent	99 (90.0%)	260 (83.1%)			65 (90.3%)	160 (86.0%)		
Pleural attachment			13.755^c^	<0.001			7.412^c^	0.006
Present	23 (20.9%)	127 (40.6%)			14 (19.4%)	69 (37.1%)		
Absent	87 (79.1%)	186 (59.4%)			58 (80.6%)	117 (62.9%)		
Spiculation			4.616^c^	0.032			2.844^c^	0.092
Present	11 (10.0%)	59 (18.8%)			7 (9.7%)	34 (18.3%)		
Absent	99 (90.0%)	254 (81.2%)			65 (90.3%)	152 (81.7%)		
Lesion-lung interface			16.517^c^	<0.001			9.119^c^	0.003
Clear	89 (80.9%)	186 (59.4%)			59 (81.9%)	116 (62.4%)		
Blurry	21 (19.1%)	127 (40.6%)			13 (18.1%)	70 (37.6%)		

^a^t value; ^b^Z value; ^c^χ^2^ value

### Interobserver and intraobserver agreements

Intraobserver and intraobserver agreements between three radiologists were near perfect, the ICC values of quantitative parameters and kappa coefficients of categorical variables were all greater than 0.75 (supplemental Tables 1 and 2).

### Screening for independent risk factors

Univariate analysis of the derivation cohort indicated lesion size (OR =1.373; 95% CI, 1.061-1.777, *P* =0.016), mean CT value (OR =1.005; 95% CI, 1.001-1.010, *P* =0.024), vascular change (OR =5.125; 95%CI, 1.437-18.281, *P* =0.012), lobulation (OR =6.196; 95%CI, 2.007-19.127, *P* =0.002), and spiculation (OR =2.436; 95%CI, 1.055-5.625, *P* =0.037) correlated with adenocarcinomas. However, volume, mass, bronchiole change, bubble, pleural attachment, and lesion-lung interface did not correlate with adenocarcinomas (*P* >0.05). Stepwise multivariate analysis showed that lesion size (OR =1.335; 95% CI, 1.178-1.512, *P* <0.001), mean CT value (OR =1.005; 95% CI, 1.002-1.008, *P* =0.002), vascular change (OR =5.771; 95% CI, 1.659-20.074, *P* =0.006), and lobulation (OR =6.528; 95% CI, 2.173-19.608, *P* =0.001) were independent risk factors for adenocarcinomas (Table 4).

**Table 4:** Univariate and multivariate logistic analysis of CT features for adenocarcinomas

**Characteristics**	**Univariate analysis**		**Multivariate analysis**	
	**OR (95%CI)**	***P* value**	**OR (95%CI)**	***P* value**
Lesion Size (mm)	1.373 (1.061-1.777)	0.016	1.335 (1.178-1.512)	<0.001
Mean CT value (HU)	1.005 (1.001-1.010)	0.024	1.005 (1.002-1.008)	0.002
Volume (cm^3^)	1.487 (0.014-153.876)	0.867		
Mass (g)	0.230 (0.000-16693.733)	0.230		
Vascular change	5.125 (1.437-18.281)	0.012	5.771 (1.659-20.074)	0.006
Bronchiole change	2.153 (0.780-5.942)	0.139		
Lobulation	6.196 (2.007-19.127)	0.002	6.528 (2.173-19.608)	0.001
Bubble	2.134 (0.931-4.891)	0.073		
Pleural attachment	0.259 (0.034-1.960)	0.259		
Spiculation	2.436 (1.055-5.625)	0.037	1.923 (0.856-4.320)	0.113
Lesion-lung interface	6.322 (0.817-48.942)	0.077		

### Construction of the nomogram model

**Fig. 3:**
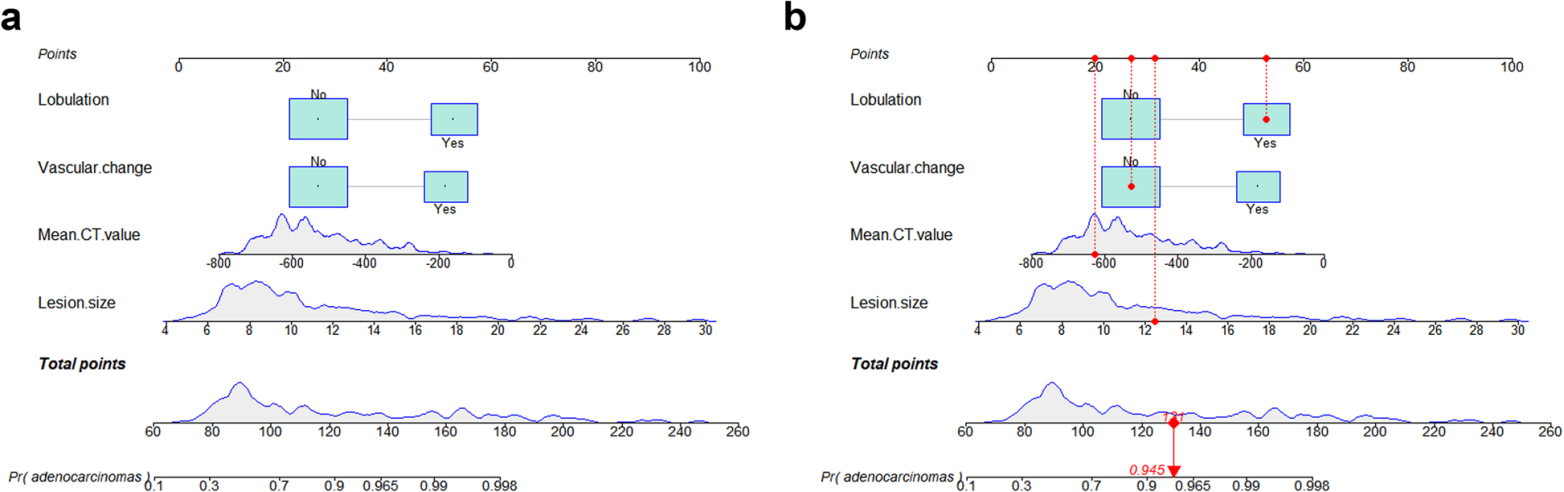
**a** A nomogram for predicting the probability of adenocarcinomas in patients with subsolid nodules (SSNs). **b** A SSN with a lesion size of 12.5mm, mean CT value -627 HU, vascular change (-), lobulation (+). The total points of SSN was 131, and the probability of adenocarcinomas was 0.945

Based on univariate and multivariate logistic regression analysis results, an individualized nomogram was generated by incorporating the 4 independent risk factors, namely lesion size, mean CT value, vascular change and lobulation (Fig. 3a). The nomogram showed that lesion size was the most important contributor to discrimination, followed by mean CT value, lobulation, and vascular change. Each independent risk factor in the nomogram was assigned a point based on regression coefficient and a straight line drawn based on total points. Finally, the probabilities of individual values were determined using the function conversion relationship of total points (Fig. 3b).

### Validation and calibration of the nomogram

**Fig. 4:**
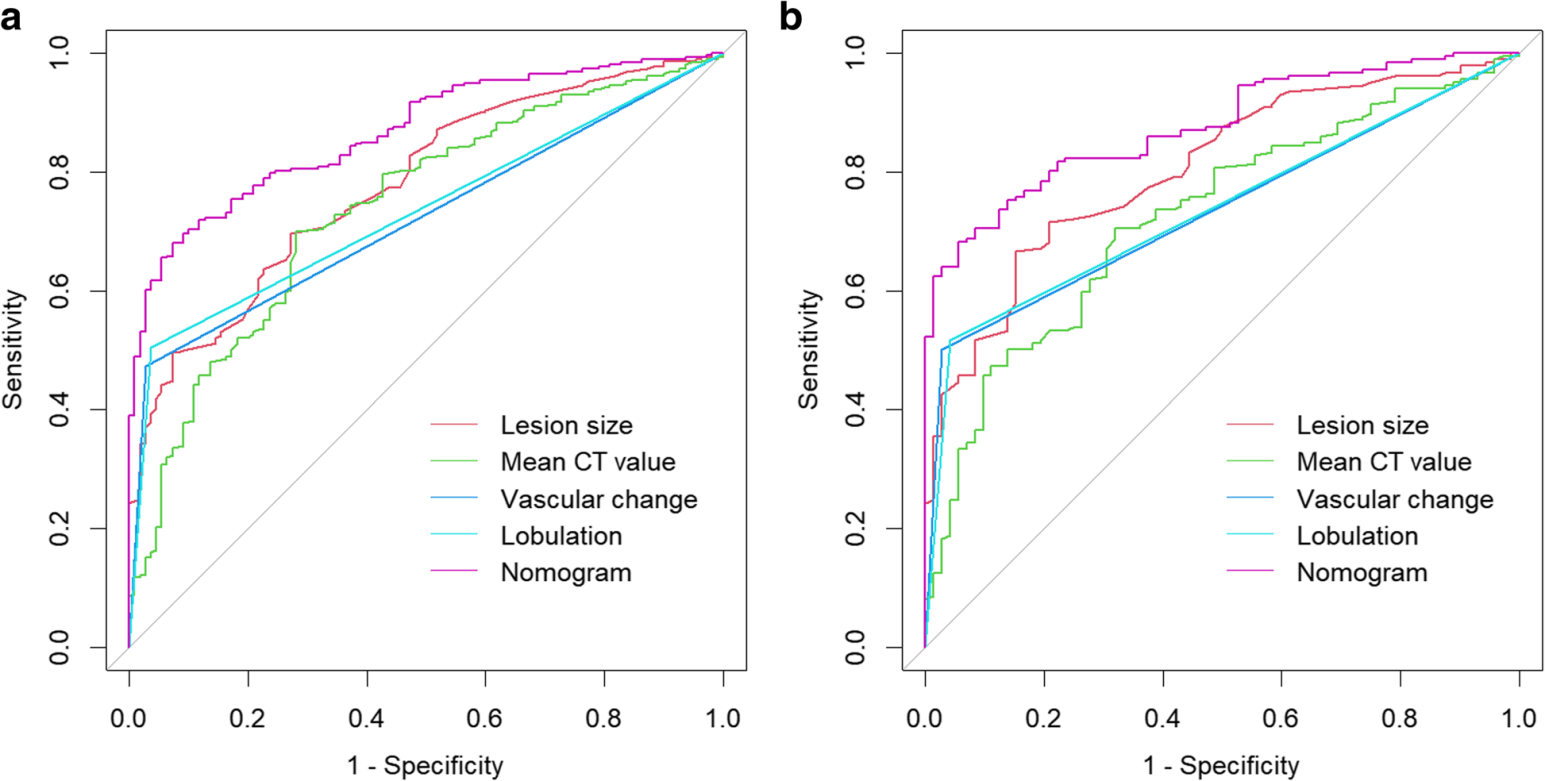
Roc curves of the nomogram and independent risk factors in the derivation cohort and validation cohort of adenocarcinomas. **a** Derivation cohort. **b** Validation cohort

In the derivation cohort, the C-index of the nomogram in predicting adenocarcinomas was 0.867 (95% CI, 0.833-0.901) which exceeded that of the lesion size (C-index =0.779; 95% CI, 0.733-0.825), mean CT value (C-index =0.740; 95% CI, 0.688-0.793), vascular change (C-index =0.723; 95% CI, 0.691-0.754), and lobulation (C-index =0.734, 95% CI, 0.701-0.767) (Fig. 4a, Table 5). Lesion size 8.5mm and mean CT value -579.5 HU were the optimal threshold values for adenocarcinomas.

**Table 5:** The C-indexes of the nomogram and variables from the logistic regression algorithm in the derivation and validation cohorts

**Characteristics**	**Derivation cohort**			**Validation cohort**		
	**C-index (95% CI)**	**Sensitivity**	**Specificity**	**C-index (95% CI)**	**Sensitivity**	**Specificity**
Nomogram model	0.867 (0.833-0.901)	0.681	0.927	0.877 (0.836-0.917)	0.683	0.944
Lesion size	0.779 (0.733-0.825)	0.696	0.727	N/A	N/A	N/A
Mean CT value	0.740 (0.688-0.793)	0.700	0.718	N/A	N/A	N/A
Vascular change	0.723 (0.691-0.754)	0.473	0.973	N/A	N/A	N/A
Lobulation	0.734 (0.701-0.767)	0.501	0.964	N/A	N/A	N/A

**Fig. 5:**
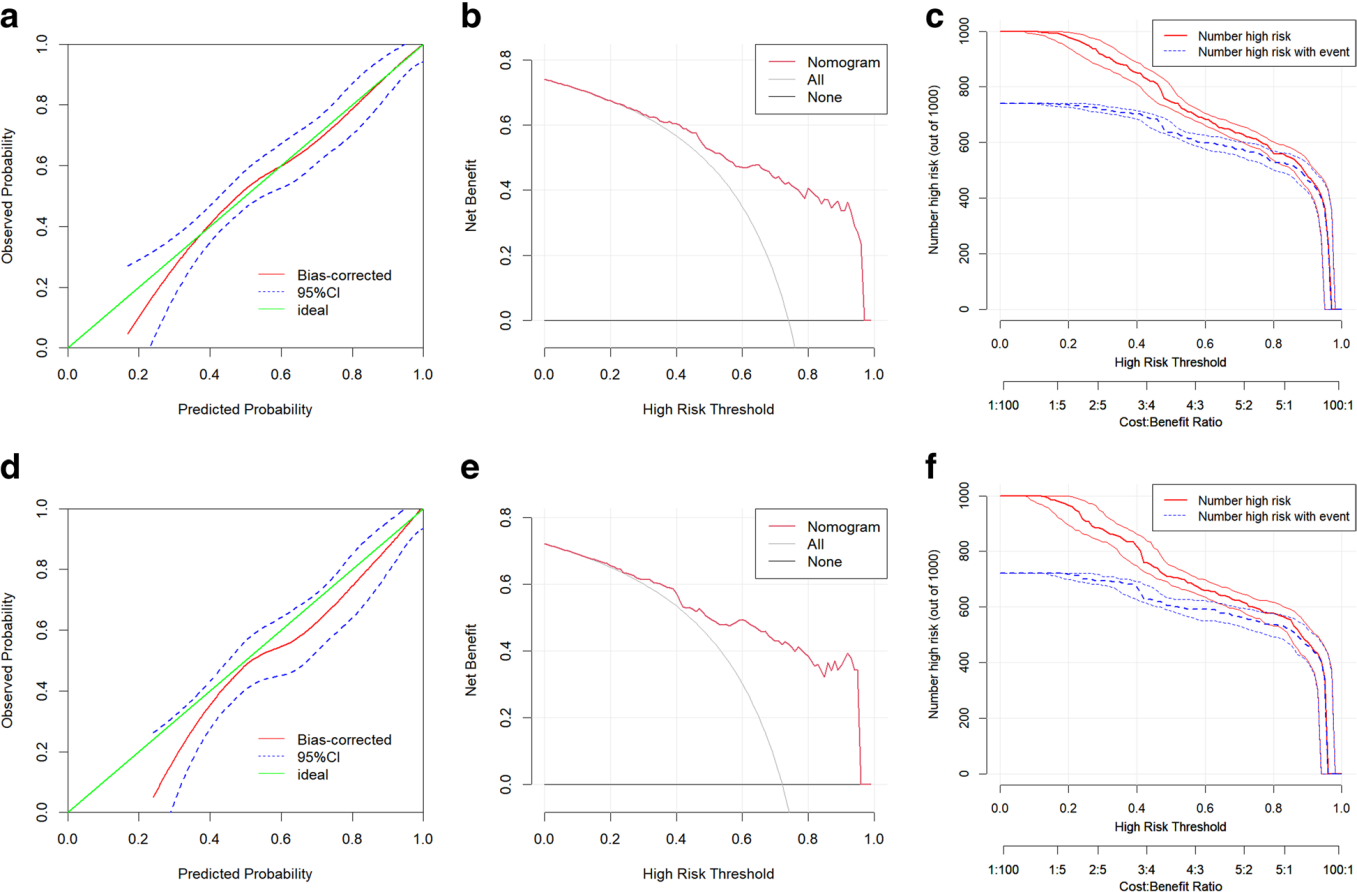
Analysis of the prediction performance of the nomogram in the **a-c** Derivation cohort and **d-f** Validation cohort. **a**, **d** Calibration curve, **b**, **e** Decision curve, **c**, **f** Clinical impact curve

Furthermore, the C-index of 0.877 (95% CI, 0.836-0.917) indicated that the nomogram had good discrimination in the validation cohort (Fig. 4b, Table 5). Evaluation of the nomograms’ performance using calibration curves, with the 45-degree line indicating best performance, revealed that the predicted results were strongly consistent with the actual results in both derivation and validation cohorts (Fig. 5a, d). Decision curve analysis of the nomogram’s value and clinical impact curve analysis revealed that the nomogram had good standardized net benefit and prediction performance (Fig. 5b, c, e, f).

## Discussion

Subsolid nodules (SSNs), including pure ground-glass nodules (GGNs) and part-solid nodules (PSNs), are common in many malignant and benign diseases such as metastatic lesions, focal fibrosis, aspergillosis, Wegener’s granulomatosis or bronchiolitis obliterans organizing pneumonia, and some SSNs are associated with lung adenocarcinoma (LUAD) or its precursors [17, 18]. The use of CT screening has increased the detection rate of LUAD [19]. However, a major challenge in CT screening is the high prevalence of SSNs but the relatively low incidence of adenocarcinomas [20], because precursor glandular lesions presenting as SSNs tends be indolent with slow growth and low metastatic potential [21–23].

CT imaging can provide accurate differentiation of various stages of LUAD progression. Jin et al. constructed a nomogram model to distinguish IAC from AAH/AIS/MIA [24]. The model can be used to determine the choice of surgical approach (IAC: standard lobectomy and the scope of lymph node dissection; AAH/AIS/MIA: sublobar resection). However, systematic studies have shown that patients with AIS may benefit from long-term follow-up, and the 2021 WHO included AAH and AIS as precursor glandular lesions and classifies MIA and IAC as adenocarcinomas. In clinical work, to reduce patient anxiety and avoid unnecessary surgery as well as reduce waste of medical resources, it is important to inform the patient whether surgical intervention is required and which lesions require priority surgery when the patient has multiple nodules. More recently, application of deep learning methods has improved lung nodule classification [25]. Jiang et al. classified SSNs on CT images based on convolutional neural networks (CNN) model [26], the model showed high accuracy. Despite that, clinicians find it difficult to determine whether surgical intervention is required based on simple and intuitive CT features. Therefore, there is a need to develop objective, unified, and standardized assessment of lesions to improve patient diagnosis and treatment. In our study, we examined simple and intuitive CT features and generated a nomogram for distinguishing adenocarcinomas from precursor glandular lesions appearing as SSNs. In the constructed nomogram, lesion size carried the highest risk of adenocarcinoma, followed by mean CT value, lobulation, and vascular change. The nomogram showed optimal discrimination and excellent calibration in the derivation and validation cohorts. The nomogram also exhibited greater net clinical benefit as revealed by decision and clinical impact curve analyses.

Lesion size has been incorporated into the Fleischner society guidelines for the management of SSNs [27]. In this study, we found that the optimal cutoff value for adenocarcinomas was 8.5 mm, which is slightly lower than that reported in previous studies [28, 29]. This discrepancy may be explained by the inclusion of SSNs compared with the inclusion of only pure GGNs in previous studies. In the clinical settings, PSNs account for a large proportion of sub-centimeter adenocarcinomas [30, 31]. It is worth mentioning that size criteria are not uniform, with several studies using the maximum diameter [32, 33]. However, the International Early Lung Cancer Action Program (iELCAP) recommends the average diameter based on long- and short-axis diameters [23, 27]. High CT value has been associated with increased lesion heterogeneity. As the lesions grow, SSNs may show increased internal density without significant lesion change [34]. Ikeda et al. reported that the mean CT value is optimal for discriminating AIS from adenocarcinomas [35]. Zhao et al. reported that average CT value is an independent risk factor for discriminating pre-invasiveness from invasiveness [30]. However, the study by Han et al. found that average CT attenuation has little significance in IAC [12]. These discrepancies may be due to the inaccuracy of the manual measurement of mean CT value. Here, we used a semi-automated tool to analyze average CT value, which may minimize potential measurement bias. Tumor biology studies indicate that neoangiogenesis or vascular remodeling is a major tumor-initiating event [13, 36]. During LUAD progression, cancer cells release various pro-angiogenic factors, including vascular endothelial growth factor (VEGF), which compensates for hypoxia by promoting neoangiogenesis or vasculature remodeling [37]. Our findings support the hypothesis that vascular change is more common in adenocarcinomas than in precursor glandular lesions [13, 38]. Lobulation results from irregular rates of cell growth in diverse directions and the different contraction forces of the internal fibrous tissues. Consistent with past studies [15, 33], we found that lobulation was significantly different between precursor glandular lesions and adenocarcinomas.

Subsequently, we compared performance of the constructed nomogram in distinguishing adenocarcinomas from precursor glandular lesions with several independent risk factors. Relative to single independent risk factors, the nomogram had a high C-index both in the derivation and validation cohorts, indicating it had good discrimination capacity. Moreover, when lesion size or mean CT value were applied alone in the derivation cohort, the sensitivity and specificity were 69.6% and 72.7% or 70.0% and 71.8%, respectively, while the nomogram showed a sensitivity of 68.1% and specificity of 92.7%. Based on these results, we concluded that morphological features, such as, vascular change and lobulation, can increase specificity of the model. Taken together, our findings highlight the potential value of CT morphology in managing patients with SSNs.

This work has some limitations. First, only patients who underwent surgery were recruited, while those who underwent conservative management were not excluded. The failure to include all patients with SSNs may have introduced selection bias. Secondly, this was a single-institution retrospective study, thus, multicenter studies are needed to independently validate our model. Additionally, although the quantitative CT features were processed using a semi-automated segmentation tool, which could better overcome manual measurement bias, large vessels and bronchi may have introduced segmentation bias. Therefore, the segmentation made in the current study may not achieve optimal accuracy suggesting that the segmentation algorithm should be further improved.

## Conclusion

Based on CT features, we have developed and validated a nomogram for predicting the risk of adenocarcinomas in patients with SSNs in light of the 2021 classification recommended by the WHO. The nomogram showed excellent discrimination and calibration results in the derivation and validation cohorts. It is expected to be a valuable pre-operation tool for identifying SSN patients who require surgical intervention.

## Supplementary Information

**Additional file 1: Supplemental Table 1.** The ICC values of quantitative parameters between three radiologists.

**Additional file 2: Supplemental Table 2.** The kappa coefficients of categorical variables between three radiologists.

## Data Availability

The datasets used and/or analysed during the current study are available from the corresponding author on reasonable request.
