# Supplementary material for: A CT-based nomogram for predicting the risk of adenocarcinomas in patients with subsolid nodule according to the 2021 WHO classification
Source: Cancer Imaging. 2022 Sep 5;22:46. doi: 10.1186/s40644-022-00483-1 (PMC9446567; doi:10.1186/s40644-022-00483-1)
Supplement: Supplementary file 1 — Additional file 1: Supplemental Table 1. The ICC values of quantitative parameters between three radiologists. [file 40644_2022_483_MOESM1_ESM.docx]

**Supplemental Table 1** The ICC values of quantitative parameters between three radiologists.

| **Characteristics** | **Intraobserver agreement (95% CI)** | | |  | **Interobserve agreement (95% CI)** | | |
| --- | --- | --- | --- | --- | --- | --- | --- |
|  | **Radiologist 1** | **Radiologist 2** | **Radiology 3** |  | **Radiologists 1 vs 2** | **Radiologists 1 vs 3** | **Radiologists 2 vs 3** |
| Lesion Size | 0.977(0.953, 0.989) | 0.974(0.945, 0.987) | 0.994(0.988, 0.997) |  | 0.968(0.948, 0.981) | 0.985(0.975, 0.991) | 0.983(0.971, 0.990) |
| Mean CT value | 0.916(0.832, 0.959) | 0.944(0.887, 0.973) | 0.990(0.979. 0.995) |  | 0.940(0.902, 0.964) | 0.957(0.930, 0.974) | 0.965(0.942, 0.979) |
| Volume | 0.910(0.820, 0.956) | 0.961(0.920, 0.981) | 0.997(0.994, 0.999) |  | 0.963(0.939, 0.978) | 0.963(0.938, 0.977) | 0.987(0.979, 0.992) |
| Mass | 0.909(0.819, 0.956) | 0.953(0.903, 0.977) | 0.994(0.987, 0.997) |  | 0.964(0.941, 0.978) | 0.966(0.943, 0.979) | 0.987(0.979, 0.992) |
