# Supplementary material for: A CT-based nomogram for predicting the risk of adenocarcinomas in patients with subsolid nodule according to the 2021 WHO classification
Source: Cancer Imaging. 2022 Sep 5;22:46. doi: 10.1186/s40644-022-00483-1 (PMC9446567; doi:10.1186/s40644-022-00483-1)
Supplement: Supplementary file 2 — Additional file 2: Supplemental Table 2. The kappa coefficients of categorical variables between three radiologists. [file 40644_2022_483_MOESM2_ESM.docx]

**Supplemental Table 2** The kappa coefficients of categorical variables between three radiologists.

| **Characteristics** | **Intraobserver agreement (95% CI)** | | |  | **Interobserve agreement (95% CI)** | | |
| --- | --- | --- | --- | --- | --- | --- | --- |
|  | **Radiologist 1** | **Radiologist 2** | **Radiology 3** |  | **Radiologists 1 vs 2** | **Radiologists 1 vs 3** | **Radiologists 2 vs 3** |
| Vascular change | 0.923(0.775, 1.000) | 0.861(0.675, 1.000) | 1.000(1.000, 1.000) |  | 0.820(0.672, 0.969) | 0.889(0.768, 1.000) | 0.930(0.834, 1.000) |
| Bronchiole change | 0.902(0.714, 1.000) | 0.841(0.629, 1.000) | 1.000(1.000, 1.000) |  | 0.784(0.608, 0.961) | 0.864(0.715, 1.000) | 0.918(0.807, 1.000) |
| Lobulation | 0.850(0.649, 1.000) | 0.930(0.794, 1.000) | 1.000(1.000, 1.000) |  | 0.892(0.773, 1.000) | 0.927(0.827, 1.000) | 0.964(0.895, 1.000) |
| Bubble | 1.000(1.000, 1.000) | 1.000(1.000, 1.000) | 1.000(1.000, 1.000) |  | 1.000(1.000, 1.000) | 1.000(1.000, 1.000) | 1.000(1.000, 1.000) |
| Pleural attachment | 1.000(1.000, 1.000) | 1.000(1.000, 1.000) | 1.000(1.000, 1.000) |  | 1.000(1.000, 1.000) | 1.000(1.000, 1.000) | 1.000(1.000, 1.000) |
| Spiculation | 0.841(0.629, 1.000) | 0.857(0.668, 1.000) | 1.000(1.000, 1.000) |  | 0.851(0.711, 0.990) | 0.923(0.819, 1.000) | 0.927(0.827, 1.000) |
| Lesion-lung interface | 1.000(1.000, 1.000) | 1.000(1.000, 1.000) | 1.000(1.000, 1.000) |  | 1.000(1.000, 1.000) | 1.000(1.000, 1.000) | 1.000(1.000, 1.000) |
